# Supplementary material for: Assessing perceived and functional health literacy among parents in Cyprus: A cross-sectional study
Source: PLoS One. 2023 Oct 11;18(10):e0292577. doi: 10.1371/journal.pone.0292577 (PMC10566705; doi:10.1371/journal.pone.0292577)
Supplement: S1 Table — (DOCX) [file pone.0292577.s001.docx]

STROBE Statement—checklist of items that should be included in reports of observational studies

|  | Item No. | Recommendation | Page  No. | Relevant text from manuscript |
| --- | --- | --- | --- | --- |
| **Title and abstract** | 1 | (*a*) Indicate the study’s design with a commonly used term in the title or the abstract | 1  2 | “Assessing perceived and functional health literacy among parents in Cyprus: a cross-sectional study”  A cross-sectional study was conducted with a convenience sample of 416 parents of children, aged 6 months to 15 years old, presenting in pediatric outpatient departments across three Cypriot cities. |
|  |  | (*b*) Provide in the abstract an informative and balanced summary of what was done and what was found | 2 | **Method:** A cross-sectional study was conducted with a convenience sample of 416 parents of children, aged 6 months to 15 years old, presenting in pediatric outpatient departments across three Cypriot cities. Participants completed the HLS-EU-Q47, a self-reported measure of HL, and the NVS (Newest Vital Sign), a performance-based measure of HL. Associations with socio-demographic characteristics and health behaviors were explored.  **Results:** Based on suggested ranges, among 416 parents, mostly mothers (83.2%), almost half of parents (42.6%) were classified as having inadequate or problematic perceived HL. Consistently, 62.8% showed high likelihood or significant possibility of limited functional HL, based on the NVS with a mean score of 2.73 out of 6 (SD=2.02). Nevertheless, no correlation was observed between the two measures of HL. Parental perceived HL was statistically significantly associated with educational attainment, number of children in the family, self-assessed health status, and exercise habits. Parental functional HL was statistically significantly associated with gender, age, education, financial aid, family income, and alcohol consumption. |
| Introduction | | | |  |
| Background/rationale | 2 | Explain the scientific background and rationale for the investigation being reported | 4 | The great majority of previous studies on HL have commonly used a single tool to measure HL focusing either on self-assessed HL or performance-based HL [18-20]. Few studies have explored in parallel both self-assessed (perceived) HL and performance-based (functional) HL. A systematic review [21] identified only four fair quality studies which explored the association between both performance-based and self-assessed HL and health outcomes with mixed results. Nevertheless, most studies included in the systematic review found a similar relationship between results of performance-based and self-assessed tools and health outcomes. Several studies since then have explored both performance-based and self-assessed HL but they still remain limited. They either measured different types of HL, such as self-assessed tool to measure eHealth Literacy, which refers to the ability to seek, find, understand, and appraise health information from electronic sources [22], and performance-based tool to measure HL [23-25]. Some others used two performance-based tools [26], or a single screening question as the second HL tool [27]. Furthermore, those studies were conducted among varied population groups other than parents. |
| Objectives | 3 | State specific objectives, including any prespecified hypotheses | 5 | The primary aim of the current study was to examine both self-assessed HL and performance-based HL among Cypriot parents. Additionally, the study explored the extent to which sociodemographic characteristics (such as educational attainment, financial status) and health-related behaviours (such as smoking, exercise habits) are potential predictors of parental perceived HL and functional HL, in order to identify a set of characteristics of a parent with limited HL. |
| Methods | | | |  |
| Study design | 4 | Present key elements of study design early in the paper | 5 | This is a descriptive study with a correlational design. |
| Setting | 5 | Describe the setting, locations, and relevant dates, including periods of recruitment, exposure, follow-up, and data collection | 5 | Study participants were recruited over a 2-year period, between January 2020 and December 2021 from three paediatric primary care practices across three different Cypriot cities; Nicosia, Limassol, and Paphos. While a convenience sampling approach was employed, the inclusion of three primary care practices aimed at capturing enough variation of the study population in terms of sociodemographic characteristics. |
| Participants | 6 | (*a*) *Cohort study*—Give the eligibility criteria, and the sources and methods of selection of participants. Describe methods of follow-up  *Case-control study*—Give the eligibility criteria, and the sources and methods of case ascertainment and control selection. Give the rationale for the choice of cases and controls  *Cross-sectional study*—Give the eligibility criteria, and the sources and methods of selection of participants | 5,6 | The inclusion criteria were: (a) parent or guardian accompanying the child to the practice, (b) older than 18 years of age, irrespective of gender (e.g. mother or father of the accompanying child), (c) the child in reference was aged six months to fifteen years old without any chronic medical condition or a history of a premature birth, (d) permanent resident of Cyprus and GeSY recipients, and (e) ability to communicate, read and write in either Greek or English to complete the questionnaire pack. Grandparents or other individuals with no parental legal responsibility accompanying the child to the primary care practice were not included.  Recruitment of study participants was performed solely by the main researcher (JM) who is a Registered Nurse with no employment or other connection with the specific primary care practices. Visits were performed 2-3 times per week and at different times a day in consecutive time periods for each practice. After informing eligible parents about the scope and process of the study, including a description of the tools and tasks that will be required from them, enrolled participants provided informed consent in writing. |
|  |  | (*b*) *Cohort study*—For matched studies, give matching criteria and number of exposed and unexposed  *Case-control study*—For matched studies, give matching criteria and the number of controls per case |  | N/A |
| Variables | 7 | Clearly define all outcomes, exposures, predictors, potential confounders, and effect modifiers. Give diagnostic criteria, if applicable | 3  7-9 | A more comprehensive definition of HL derived from the review of 17 conceptual frameworks and definitions [12] is that HL “entails people’s knowledge, motivation and competences to access, understand, appraise, and apply health information in order to make judgments and take decisions in everyday life concerning healthcare, disease prevention and health promotion to maintain or improve quality of life during the life course”.  Participants were also asked to provide basic sociodemographic information, including age, gender, marital status, nationality, place of residence (urban vs rural), and other, expected to demonstrate an association with HL based on previous studies [28-29]. Furthermore, in order to explore the potential social gradient in parental HL and in the absence of a standard measure of socioeconomic status in Cyprus, a range of variables was explored including educational attainment, monthly family income, employment status, financial difficulties (e.g. paying bills in the past 12 months), and/or receipt of financial aid. Finally, the subjective perception of relative standing on the social hierarchy was measured using a variation of the MacArthur Scale [33]. Such measures are considered particularly relevant as they represent an internalized perception of social position through a process of social comparison. Specifically, the measure represents the social structure as a ladder with 10 steps, where responders locate themselves on the ladder considering that the top represents people who are best-off (most privileged in terms of education, income, and employment) and the bottom people who are worst-off (most disadvantaged).  Parents also provided information on a range of health-related behaviours, such as smoking habits, alcohol consumption, and provided a standard self-assessment of their own health status on a five-point scale ranging from poor to excellent. |
| Data sources/ measurement | 8* | For each variable of interest, give sources of data and details of methods of assessment (measurement). Describe comparability of assessment methods if there is more than one group | 7-9 | Perceived HL was assessed using the Greek-version of the HLS-EU-Q47 questionnaire. This is one of the most widely used measures of HL, originally developed in the context of a European project across eight countries, namely Austria, Bulgaria, Germany, Greece, Ireland, Netherlands, Poland, and Spain [29]. The tool consists of 47 items measuring perceived HL with regard to three domains: healthcare (16 questions), disease prevention (15 questions), and health promotion (16 questions). In all domains, items tap on four competences that influence a person’s decision making: accessing, understanding, appraising, and applying health information. The answers are given on a four-point Likert response scale whereby 1 corresponds to “very difficult” to 4 to “very easy”. The total score is expressed in the 0 to 50 range, whereby score <26 indicates inadequate, 26-33 problematic, 34-42 adequate HL, and 43-50 excellent perceived HL. The tool has been shown to have good reliability and validity [19, 30-32]. In regard to the Cypriot population, a pilot validity assessment of the Greek translated version of the tool showed good metric properties and was able to capture the expected social gradient in HL by indicators of socio-economic disadvantage [28].  Functional HL was assessed using the NVS tool. The NVS was developed by Pfizer Inc in English and Spanish to measure functional HL. It is a task-based measure of HL based on an ice cream nutrition label with six related questions assessing prose literacy, document literacy, and numeracy skills. The total score ranges from 0 to 6. A score of 0 and 1 suggests high likelihood of limited HL, score of 2 and 3 suggests the possibility of limited HL, and score between 4 and 6 suggests adequate HL. The tool was reported to have good internal consistency with Cronbach a >0.76 and has been validated against other tools that measure functional HL, such as TOFHLA [16].  Participants were also asked to provide basic sociodemographic information, including age, gender, marital status, nationality, place of residence (urban vs rural), and other, expected to demonstrate an association with HL based on previous studies [28-29]. Furthermore, in order to explore the potential social gradient in parental HL and in the absence of a standard measure of socioeconomic status in Cyprus, a range of variables was explored including educational attainment, monthly family income, employment status, financial difficulties (e.g. paying bills in the past 12 months), and/or receipt of financial aid. Finally, the subjective perception of relative standing on the social hierarchy was measured using a variation of the MacArthur Scale [33]. Such measures are considered particularly relevant as they represent an internalized perception of social position through a process of social comparison. Specifically, the measure represents the social structure as a ladder with 10 steps, where responders locate themselves on the ladder considering that the top represents people who are best-off (most privileged in terms of education, income, and employment) and the bottom people who are worst-off (most disadvantaged).  Parents also provided information on a range of health-related behaviours, such as smoking habits, alcohol consumption, and provided a standard self-assessment of their own health status on a five-point scale ranging from poor to excellent. |
| Bias | 9 | Describe any efforts to address potential sources of bias |  | N/A |
| Study size | 10 | Explain how the study size was arrived at | 6 | The minimum sample size was set to 369. This was based on precision and power analysis using estimates from a previous study in the Cypriot population which used the HLS-EU-Q47 [28]. In that study, the percentage of healthcare users aged 30-44 years old with inadequate or problematic HL was estimated at 39.6%, while the observed standard deviation in HL scores was in the magnitude of 8. Thus, precision analysis suggests that the sample size allows the estimation of 95% confidence intervals for proportions with ±5% statistical error in the full sample. Furthermore, different power analysis scenarios suggest that the sample size is adequate to identify a moderate effect size (e.g. a difference in the magnitude of 0.25-0.30 SD between groups of interest) with 90% statistical power at the 5% level. |

Continued on next page

| Quantitative variables | 11 | Explain how quantitative variables were handled in the analyses. If applicable, describe which groupings were chosen and why | 9 | All variables of sociodemographic characteristics, and health behaviors were treated as categorical variables, except from smoking pack-years.  Pack years was addressed as the total measurement which combines the number of cigarettes that a person smokes on a daily basis (in a packet of 20 cigarettes) with the total period that the person smokes.  The outcome variables of perceived HL and functional HL were operationalized as both categorical and continuous variables. |
| --- | --- | --- | --- | --- |
| Statistical methods | 12 | (*a*) Describe all statistical methods, including those used to control for confounding | 9,10 | Descriptive statistics, including frequency (n), percentage (%), mean (M), and standard deviation (SD) were used to describe parental sociodemographic characteristics, health behaviors, perceived HL, and functional HL.  Furthermore, Pearson correlation was used to determine the strength and direction of a linear relationship between HLS-EU-Q47 score and NVS score.  To examine bivariable associations between the continuous outcome variables (perceived HL and functional HL) and predictor variables (sociodemographic characteristics, health behaviors), a series of independent sample t-tests and one-way Analysis of Variance (ANOVAs) were conducted. To examine whether there was a relationship between outcome variables (perceived HL, functional HL) treated as categorical variables, and predictor variables, chi-square tests for independence (χ2) were used. Games-Howell post-hoc analysis or Tukey post-hoc analysis was conducted to examine whether the differences between groups were statistically significant.  Stepwise multiple linear regression analyses were used to identify the strongest associations between parental perceived HL or functional HL scores with all predictor variables: parental sociodemographic characteristics and parental health behaviors after mutually adjusting for each other. The strength of the association between predictor variables and dependent variables was assessed by computing standardized regression coefficients (tiny ≤0.05, very small 0.05 to 0.10, small 0.10 to 0.20, medium 0.20 to 0.30, large 0.30 to 0.40, and very large ≥0.40) [34] and Cramer’s V (weak from 0.10 to 0.30, medium from 0.40 to 0.50, and strong >0.50) as effect sizes. Statistical significance was defined as *p*<0.05. All statistical analysis were performed using SPSS version 26. |
|  |  | (*b*) Describe any methods used to examine subgroups and interactions | 9,10 | Furthermore, Pearson correlation was used to determine the strength and direction of a linear relationship between HLS-EU-Q47 score and NVS score.  To examine bivariable associations between the continuous outcome variables (perceived HL and functional HL) and predictor variables (sociodemographic characteristics, health behaviors), a series of independent sample t-tests and one-way Analysis of Variance (ANOVAs) were conducted. To examine whether there was a relationship between outcome variables (perceived HL, functional HL) treated as categorical variables, and predictor variables, chi-square tests for independence (χ2) were used.  Stepwise multiple linear regression analyses were used to identify the strongest associations between parental perceived HL or functional HL scores with all predictor variables: parental sociodemographic characteristics and parental health behaviors after mutually adjusting for each other. |
|  |  | (*c*) Explain how missing data were addressed |  | -- |
|  |  | (*d*) *Cohort study*—If applicable, explain how loss to follow-up was addressed  *Case-control study*—If applicable, explain how matching of cases and controls was addressed  *Cross-sectional study*—If applicable, describe analytical methods taking account of sampling strategy |  | N/A |
|  |  | (*e*) Describe any sensitivity analyses |  | -- |
| Results | | | | |
| Participants | 13* | (a) Report numbers of individuals at each stage of study—eg numbers potentially eligible, examined for eligibility, confirmed eligible, included in the study, completing follow-up, and analysed | 10 | The majority among 416 participating parents |
|  |  | (b) Give reasons for non-participation at each stage |  | N/A |
|  |  | (c) Consider use of a flow diagram |  | N/A |
| Descriptive data | 14* | (a) Give characteristics of study participants (eg demographic, clinical, social) and information on exposures and potential confounders | 10,11 | The majority among 416 participating parents were female (n=346, 83.2%), married or in partnership (n=358, 86.1%), of Cypriot nationality (n=380, 91.3%) and resident in an urban area (n=285, 68.5%). Regarding parental age, almost one in three parents (n=129, 31.0%) were 30-34 years old. Almost half of them had two children (n=193, 46.4%), and seven to ten parents were working full time (n=290, 69.7%). The monthly family income in half of participants was under 2400 euro (n=210, 50.4%). Almost seven in ten reported no financial difficulties (n=285, 68.5%) and about half (n=219, 52.6%) reported that they did not receive any financial allowance. Finally, in terms of subjective social status, 14.2% of participants placed themselves at the bottom steps (1-4) of the ladder and only 10.8% on the top steps (8-10). The majority perceived themselves being between the sixth and seventh step, indicating middle-to-high social status (n=177, 42.6%). Finally, a high percentage of participants were University graduates with a bachelor or postgraduate degree (n=286, 68.7%). The socio-demographic profile of participants is presented in Table 1. |
|  |  | (b) Indicate number of participants with missing data for each variable of interest |  | -- |
|  |  | (c) *Cohort study*—Summarise follow-up time (eg, average and total amount) |  | N/A |
| Outcome data | 15* | *Cohort study*—Report numbers of outcome events or summary measures over time |  |  |
|  |  | *Case-control study—*Report numbers in each exposure category, or summary measures of exposure |  |  |
|  |  | *Cross-sectional study—*Report numbers of outcome events or summary measures |  | N/A |
| Main results | 16 | (*a*) Give unadjusted estimates and, if applicable, confounder-adjusted estimates and their precision (eg, 95% confidence interval). Make clear which confounders were adjusted for and why they were included | 22 | Table 6 |
|  |  | (*b*) Report category boundaries when continuous variables were categorized | 7,8 | The total score is expressed in the 0 to 50 range, whereby score <26 indicates inadequate, 26-33 problematic, 34-42 adequate HL, and 43-50 excellent perceived HL.  The total score ranges from 0 to 6. A score of 0 and 1 suggests high likelihood of limited HL, score of 2 and 3 suggests the possibility of limited HL, and score between 4 and 6 suggests adequate HL. |
|  |  | (*c*) If relevant, consider translating estimates of relative risk into absolute risk for a meaningful time period |  | N/A |

Continued on next page

| Other analyses | 17 | Report other analyses done—eg analyses of subgroups and interactions, and sensitivity analyses | 9,10 | To examine bivariable associations between the continuous outcome variables (perceived HL and functional HL) and predictor variables (sociodemographic characteristics, health behaviors), a series of independent sample t-tests and one-way Analysis of Variance (ANOVAs) were conducted. To examine whether there was a relationship between outcome variables (perceived HL, functional HL) treated as categorical variables, and predictor variables, chi-square tests for independence (χ2) were used.  Stepwise multiple linear regression analyses were used to identify the strongest associations between parental perceived HL or functional HL scores with all predictor variables: parental sociodemographic characteristics and parental health behaviors after mutually adjusting for each other. |
| --- | --- | --- | --- | --- |
| Discussion | | | | |
| Key results | 18 | Summarise key results with reference to study objectives | 23 | The results of the current study have shown that a high proportion of parents in Cyprus may have inadequate or problematic HL, according to both self-assessed and performance-based HL tools. Higher educational attainment, higher number of children, better health self-rated status, and more frequent exercise were statistically significantly associated with higher scores of parental perceived HL, whereas male gender, older age, higher educational attainment, not receiving financial aid, higher monthly family income, and alcohol consumption were statistically significantly associated with higher scores of parental functional HL. The combination of self-rated health status and number of children in the family were observed as important predictors of parental perceived HL, whereas the combination of self-rated health status, parental education, age, social status, and alcohol consumption as important predictors of parental functional HL. |
| Limitations | 19 | Discuss limitations of the study, taking into account sources of potential bias or imprecision. Discuss both direction and magnitude of any potential bias | 27,28 | The current study has several limitations. First, a stratified sampling may be more appropriate rather than the convenience sampling used in this study which may led to selection bias. However, convenience sampling was more feasible during the Covid-19 pandemic period, in which data collection took place, because of the restrictions in paediatric clinical settings. Additionally, a high percentage of participants were University graduates with a bachelor or postgraduate degree (n=286, 68.7%), which is not in agreement with the Cyprus population census 2011, in which the proportion of people in the participants’ age groups who had university degree (bachelor or postgraduate) was approximately 27.8%. This concludes a possible selection bias towards higher educational attainment. Finally, the cross-sectional nature of the design, disallows any causal conclusions. Therefore, a longitudinal design should be implemented in future studies. |
| Interpretation | 20 | Give a cautious overall interpretation of results considering objectives, limitations, multiplicity of analyses, results from similar studies, and other relevant evidence | 28,29 | Most studies examine separately perceived HL and functional HL. This study provides information for both perceived and functional HL in a sample of Cypriot parents and examines their association with sociodemographic characteristics and health behaviors. A substantial percentage of parents have low HL, and this conclusion is consistent irrespective of whether a perceived or functional measure is used. Nevertheless, from a large number of variables investigated, indicators of social gradient were found to be statistically significantly associated with HL. While self-rated health was associated with HL, the association between HL and health-related behaviours did not appear to show a clear pattern. Findings suggest that some parental characteristics may play an important role as predictors of their limited HL. This could imply important information in identifying parents at risk for limited HL levels to appropriately intervene and improve not only parental HL, but also consequently health and wellbeing in pediatric population. |
| Generalisability | 21 | Discuss the generalisability (external validity) of the study results |  | -- |
| Other information | |  | | |
| Funding | 22 | Give the source of funding and the role of the funders for the present study and, if applicable, for the original study on which the present article is based |  | N/A |

*Give information separately for cases and controls in case-control studies and, if applicable, for exposed and unexposed groups in cohort and cross-sectional studies.

**Note:** An Explanation and Elaboration article discusses each checklist item and gives methodological background and published examples of transparent reporting. The STROBE checklist is best used in conjunction with this article (freely available on the Web sites of PLoS Medicine at http://www.plosmedicine.org/, Annals of Internal Medicine at http://www.annals.org/, and Epidemiology at http://www.epidem.com/). Information on the STROBE Initiative is available at www.strobe-statement.org.
