# Supplementary material for: Assessing perceived and functional health literacy among parents in Cyprus: A cross-sectional study
Source: PLoS One. 2023 Oct 11;18(10):e0292577. doi: 10.1371/journal.pone.0292577 (PMC10566705; doi:10.1371/journal.pone.0292577)
Supplement: S1 Appendix — (PDF) [file pone.0292577.s002.pdf]

Dear Healthcare Professional:

Thank you for your interest in the Newest Vital Sign (NVS), the first tool available to assess health literacy in English and Spanish.

Research shows that patients with low health literacy are less likely to comply with prescribed treatment and medical instructions from their physician. Identifying patients who are at risk for low health literacy allows physicians to apply specific clear health communication techniques that may enhance understanding. The Newest Vital Sign is a simple and fast way to identify those patients. The tool, which tests literacy skills for both numbers and words*, has been validated against a previously validated measure of health literacy (the TOFHLA), and has been shown to take approximately three minutes to administer.

In addition to the NVS tool, we are also including information to help enhance patient-provider communication. In this folder you will find the following materials:

- NVS Tool (nutrition label and scoring sheet tear-off pad, both two-sided in English/Spanish)
- NVS Implemenation Guide
- *Ask Me 3* (fact sheet on free educational materials from the non-profit Partnership for Clear Health Communication)
- *Help Your Patients Succeed* (tips for improving communication with your patients)
- *Why Does An Ice Cream Label Work* . . . (fact sheet explaining the design of the NVS)

The Newest Vital Sign is Pfizer Inc's most recent contribution to the health literacy movement. For more than nine years, Pfizer has been committed to raising awareness of developing solutions for low health literacy. The overall goal of our Clear Health Communication Initiative is to positively impact the health care system by enhancing patient-provider communication to increase compliance and improve patient health outcomes.

The Newest Vital Sign and companion materials are available to medical and public health providers at no cost. To learn more about our efforts to improve health literacy, please visit www.pfizerhealthliteracy.com.

Sincerely,

Richard C. Hubbard, M.D.
Senior Director, External Medical Affairs
Pfizer Inc

*Literacy is defined as the understanding and application of words (prose), numbers (numeracy), and forms, etc. (document).

Implementation Guide for the Newest Vital Sign

Health literacy— the ability to read, understand and act upon health information — is now known to be vital to good patient care and positive health outcomes. According to the Institute of Medicine's groundbreaking report on health literacy, nearly half of all American adults — 90 million people — have difficulty understanding and using health information. When patients lack the ability to understand and act upon medical information, it can put their health at risk.

The Newest Vital Sign is a new tool designed to quickly and simply assess a patient's health literacy skills. It can be administered in only 3 minutes and is available in English and Spanish. The patient is given a specially designed ice cream nutrition label to review and is asked a series of questions about it. Based on the number of correct answers, health care providers can assess the patient's health literacy level and adjust the way they communicate to ensure patient understanding.

There are many ways to integrate the Newest Vital Sign (NVS) into a private practice or clinic setting to improve communication with patients. Improved communication can help increase your patients' ability to understand and act upon the information you provide; ultimately improving patient satisfaction and health outcomes.

How To Use the Newest Vital Sign

1. **Who and when to administer the Newest Vital Sign.**
   - **A nurse (or other trained clinic staff)** is the preferred administrator of the Newest Vital Sign.
   - Administer at the same time that other vital signs are being taken.
2. **Ask the patient to participate.**
   A useful way to ask the patient is an explanation similar to this:
   > *"We are asking our patients to help us learn how well patients can understand the medical information that doctors give them. Would you be willing to help us by looking at some health information and then answering a few questions about that information? Your answers will help our doctors learn how to provide medical information in ways that patients will understand. It will only take about 3 minutes."*
3. **Hand the nutrition label to the patient.**
   The patient can and should retain the nutrition label throughout administration of the Newest Vital Sign. The patient can refer to the label as often as desired.

More…

4. **Start Asking the 6 questions, one by one, giving the patient as much time as needed to refer to the nutrition label to answer the questions.**
    - There is no maximum time allowed to answer the questions. The average time needed to complete all 6 questions is about 3 minutes. However, if a patient is still struggling with the first or second question after 2 or 3 minutes, the likelihood is that the patient has limited literacy and you can stop the assessment.
    - **Ask the questions in sequence.** Continue even if the patient gets the first few questions wrong. However, **if question 5 is answered incorrectly, do not ask question 6.**
    - **You can stop asking questions if a patient gets the first four correct.** With four correct responses, the patient almost certainly has adequate literacy.
    - **Do not prompt patients who are unable to answer a question.** Prompting may jeopardize the accuracy of the test. Just say, "Well, then let's go on to the next question."
    - **Do not show the score sheet to patients.** If they ask to see it, tell them that "I can't show it to you because it contains the answers, and showing you the answers spoils the whole point of asking you the questions."
    - **Do not tell patients if they have answered correctly or incorrectly.** If patients ask, say something like: "I can't show you the answers till you are finished, but for now you are doing fine. Now let's go on to the next question."
5. **Score by giving 1 point for each correct answer (maximum 6 points).**
    - **Score of 0-1** suggests high likelihood (50% or more) of limited literacy.
    - **Score of 2-3** indicates the possibility of limited literacy.
    - **Score of 4-6** almost always indicates adequate literacy.

    Record the NVS score in the patient's medical record, preferably near other vital sign measures.

Best Practices for Implementation: Summary

- A nurse (or other trained clinic staff) is the preferred administrator of the Newest Vital Sign.
- Administer the NVS at the same time that the patient's other vital signs are being taken.
- Record the NVS score in the patient's chart, preferably near other vital sign measures.
- Tailor communication to ensure patient understanding.

Why Does an Ice Cream Label Work as a Predictor of the Ability To Understand Medical Instructions?

A patient's ability to read and analyze any kind of nutrition label requires the same analytical and conceptual skills that are needed to understand and follow a provider's medical instructions. The skills, which are known as *health literacy,* are defined as the understanding and application of words (prose), numbers (numeracy), and forms (documents).

The use of an ice cream label is especially relevant as recent research in the *American Journal of Preventive Medicine* (November 2006) has shown that poor comprehension of food labels correlated highly with low-level literacy and numeracy skills. However, the study found that even patients with better reading skills could have difficulties interpreting the labels.

Whether reading a food label or following medical instructions, patients need to:

- remember numbers and make mathematical calculations.
- identify and be mindful of different ingredients that could be potentially harmful to them.
- make decisions about their actions based on the given information.

PROSE LITERACY:
Clinical example: The patient has scheduled some blood tests and is instructed in writing to fast the night before the tests. The skill needed to follow this instruction is **Prose Literacy.**

Ice cream label example: The patient needs this skill to read the label and determine if he can eat the ice cream if he is allergic to peanuts.

NUMERACY:
Clinical example: A patient is given a prescription for a new medication that needs to be taken at a certain dosage twice a day. The skill needed to take the medication properly is **Numeracy.**

Ice cream label example: The patient needs this same skill to calculate how many calories are in a serving of ice cream.

DOCUMENT LITERACY:
Clinical example: The patient is told to buy a glucose meter and use it 30 minutes before each meal and before going to bed. If the number is higher than 200, he should call the office. The skill needed to follow this instruction is **Document Literacy.**

Ice cream label example: The patient needs this skill to identify the amount of saturated fat in a serving of ice cream and how it will affect his daily diet if he doesn't eat it.

Nutrition Facts

Serving Size			½ cup
Servings per container			4
Amount per serving			
Calories	250	Fat Cal	120
			%DV
**Total Fat** 13g			20%
Sat Fat 9g			40%
**Cholesterol** 28mg			12%
**Sodium** 55mg			2%
**Total Carbohydrate** 30g			12%
Dietary Fiber 2g			
Sugars 23g			
**Protein** 4g			8%

*Percentage Daily Values (DV) are based on a 2,000 calorie diet. Your daily values may be higher or lower depending on your calorie needs.

Ingredients: Cream, Skim Milk, Liquid Sugar, Water, Egg Yolks, Brown Sugar, Milkfat, Peanut Oil, Sugar, Butter, Salt, Carrageenan, Vanilla Extract.

The Newest Vital Sign
A Health Literacy Assessment Tool

Score Sheet for the Newest Vital Sign Questions and Answers

READ TO SUBJECT:
This information is on the back of a container of a pint of ice cream.

1. If you eat the entire container, how many calories will you eat?
   Answer: *1,000 is the only correct answer*

2. If you are allowed to eat 60 grams of carbohydrates as a snack, how much ice cream could you have?
   Answer: *Any of the following is correct: 1 cup (or any amount up to 1 cup), half the container. Note: If patient answers "two servings," ask "How much ice cream would that be if you were to measure it into a bowl?"*

3. Your doctor advises you to reduce the amount of saturated fat in your diet. You usually have 42 g of saturated fat each day, which includes one serving of ice cream. If you stop eating ice cream, how many grams of saturated fat would you be consuming each day?
   Answer: *33 is the only correct answer*

4. If you usually eat 2,500 calories in a day, what percentage of your daily value of calories will you be eating if you eat one serving?
   Answer: *10% is the only correct answer*

READ TO SUBJECT:
Pretend that you are allergic to the following substances: penicillin, peanuts, latex gloves, and bee stings.

5. Is it safe for you to eat this ice cream?
   Answer: No

6. (Ask only if the patient responds "no" to question 5): Why not?
   Answer: *Because it has peanut oil.*

ANSWER CORRECT?	yes	no
1		
2		
3		
4		
5		
6		
Number of correct answers:		

Interpretation

Score of 0-1 suggests high likelihood (50% or more) of limited literacy.
Score of 2-3 indicates the possibility of limited literacy.
Score of 4-6 almost always indicates adequate literacy.
