# Supplementary material for: Assessing perceived and functional health literacy among parents in Cyprus: A cross-sectional study
Source: PLoS One. 2023 Oct 11;18(10):e0292577. doi: 10.1371/journal.pone.0292577 (PMC10566705; doi:10.1371/journal.pone.0292577)
Supplement: S2 Appendix — (PDF) [file pone.0292577.s003.pdf]

HLS-EU-Q47

©HLS-EU Consortium

**Item no.**	**On a scale from very easy to very difficult, how easy would you say it is to:…**	**1 Very Difficult**	**2 Difficult**	**3 Easy**	**4 Very Easy**	5 (Don't know- to be used by interviewer only)
1	Find information about symptoms of illnesses that concern you?					
2	Find information on treatments of illnesses that concern you?					
3	Find out what to do in case of a medical emergency?					
4	Find out where to get professional help when you are ill?					
5	Understand what your doctor says to you?					
6	Understand the leaflets that come with your medicine?					
7	Understand what to do in a medical emergency?					
8	Understand your doctor's or pharmacist's instruction on how to take a prescribed medicine?					
9	Judge how information from your doctor applies to you?					
10	Judge the advantages and disadvantages of different treatment options?					
11	Judge when you may need to get a second opinion from another doctor?					
12	Judge if the information about illness in the media is reliable?					
13	Use information the doctor gives you to make decisions about your illness?					
14	Follow the instructions on medication?					
15	Call an ambulance in an emergency?					
16	Follow instructions from your doctor or pharmacist?					

Item no.	On a scale from very easy to very difficult, how easy would you say it is to:…	1 Very difficult	2 Difficult	3 Easy	4 Very easy	5 (Don’t know- to be used by interviewer only)
17	Find information about how to manage unhealthy behavior such as smoking, low physical activity and drinking too much?					
18	Find information on how to manage mental health problems like stress or depression?					
19	Find information about vaccinations and health screenings that you should have?					
20	Find information on how to prevent or manage conditions like being overweight, high blood pressure or high cholesterol?					
21	Understand health warnings about behavior such as smoking, low physical activity and drinking too much?					
22	Understand why you need vaccinations?					
23	Understand why you need health screenings?					
24	Judge how reliable health warnings are, such as smoking, low physical activity and drinking too much?					
25	Judge when you need to go to a doctor for a check-up?					
26	Judge which vaccinations you may need?					
27	Judge which health screenings you should have?					
28	Judge if the information on health risks in the media is reliable?					
29	Decide if you should have a flu vaccination?					
30	Decide how you can protect yourself from illness based on advice from family and friends?					

Item no.	On a scale from very easy to very difficult, how easy would you say it is to:…	1 Very difficult	2 Difficult	3 Easy	4 Very easy	5 (Don't know- to be used by interviewer only)
31	Decide how you can protect yourself from illness based on information in the media?					
32	Find information on healthy activities such as exercise, healthy food and nutrition?					
33	Find out about activities that are good for your mental well-being?					
34	Find information on how your neighborhood could be more health-friendly?					
35	Find out about political changes that may affect health?					
36	Find out about efforts to promote your health at work?					
37	Understand advice on health from family members or friends?					
38	Understand information on food packaging?					
39	Understand information in the media on how to get healthier?					
40	Understand information on how to keep your mind healthy?					
41	Judge where your life affects your health and well-being?					
42	Judge how your housing conditions help you to stay healthy?					
43	Judge which everyday behavior is related to your health?					
44	Make decisions to improve your health?					
45	Join a sports club or exercise class if you want to?					
46	Influence your living conditions that affect your health and wellbeing?					
47	Take part in activities that improve health and well-being in your community?					
