# Supplementary material for: Assessing perceived and functional health literacy among parents in Cyprus: A cross-sectional study
Source: PLoS One. 2023 Oct 11;18(10):e0292577. doi: 10.1371/journal.pone.0292577 (PMC10566705; doi:10.1371/journal.pone.0292577)

General Demographic Data

Please choose the data which represents you most in each question, by putting the symbol X in the suitable square.

Child Information				
**Gender**	Male		**Child’s number in the family**	
	Female		**Child’s Weight**	
**Date of Birth**			**Child’s Height**	

**1. Gender**	Male		**2. Place of Living**	City area	
	Female			Village	

**3. Age**	<20		40-44		**4.How many children do you have?**	1	
	20-24		45-59			2	
	25-29		50-54			3	
	30-34		55-59			≥4	
	35-39		≥60				

**5. Nationality**	Cyprus		Other………… *(please specify)*		If other, how long have you been in Cyprus?	

**6.Marital Status**	Married		**7. Educational Level**	Not attend any school at all/Attended some years of Primary School	
	Not Married			Primary School	
	Divorced			Completed 3rd grade of Secondary School	
	Widowed			Completed Secondary School	
	In partnership (living together)			College	
				University	
				Postgraduate Studies	

**8. Do you receive any financial help;**	YES		**9. Do you face any financial difficulty in your daily needs/ bill payments during the last 12 months?**	YES	
	NO			NO	

---

10. How often do you exercise for 30 minutes or more, for example running, walking, cycling, during the last month?

Almost every day	
Some times per week	
Some times per month	
Never	
I could not exercise	

11. In regard to smoking cigarette, cigar, or pipe, which of the below statements apply to you?

I smoke	
I used to smoke in the past, but I stopped it	
I have never smoked in the past	

12. If you smoke, please answer the questions below:

12.1. For how many years do you smoke? ……………………..
12.2. How many cigarettes do you smoke daily? ……………………….

13. If you used to smoke in the past, please answer the questions below:

13.1. For how many years did you smoke in total? ……………………..
13.2. How many cigarettes did you smoke daily? …………………….

14. Have you drunk any alcoholic drink (beer, wine) during the last 30 days?

YES	
NO	

15. If you answer YES, how many times have you drunk an alcoholic drink during the last 30 days?

Every day	
4-5 timer per week	
2-3 times per week	
Once a week	
2-3 times per month	
1 time	

16. How will you evaluate your health in general?

Very good	
Good	
Moderate	
Bad	
Very bad	

17. How many times have you visited the doctor during the last 12 months?

None	
1-2 times	
3-5 times	
6-8 times	
At least 8 times	

18. What is the monthly salary of your family?

Under €800	
€800 - €1,349	
€1,350 - €1,849	
€1,850 - €2,399	
€2,400 - €2,949	
€2,950 - €3,599	
€3,600 - €4,399	
€4,400 - €5,249	
€5,250 - €6,449	
€6,450 - €7,449	
€7,450 - €8,449	
Πάνω από €8,450	

19. What is your current employment status?

Full time	0
Part time	1
Unemployed	2
Student, work experience with no salary	3
Retired	4
Permanent disability	5
Temporary work disability due to a disease	6
Military or social service	7
Home, carer	8
Other (REPORT) ………………………………………………	

20. Look at the below scale

Think that in the top of the scale, there are people who have the most money, the highest education, and the more prestige works. In the bottom of the scale, there are people who have the lowest amount of money, the lowest education, and the works with the lowest prestige or they do not have job at all.

If you think your own situation (salary, education, and job), where will you put yourself in the scale in relation to the other people?

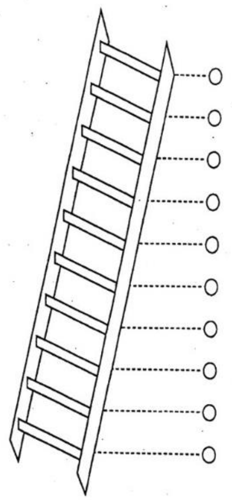

Supplement: S3 Appendix — (PDF) [file pone.0292577.s004.pdf]
